# Analysis of red autofluorescence (650-670nm) in epidermal cell populations and its potential for distinguishing contributors to 'touch' biological samples

**DOI:** 10.12688/f1000research.8036.1

**Published:** 2016-02-16

**Authors:** Cristina E. Stanciu, M. Katherine Philpott, Eduardo E. Bustamante, Ye Jin Kwon, Christopher J. Ehrhardt

**Affiliations:** 1 Department of Forensic Science, Virginia Commonwealth University, Richmond, VA, USA

**Keywords:** forensic science, flow cytometry, epidermal cell, touch DNA, autofluorescence, mixture

## Abstract

Interpretation of touch DNA mixtures poses a significant challenge for forensic caseworking laboratories.  Front end techniques that facilitate separation of contributor cell populations before DNA extraction are a way to circumvent this problem. The goal of this study was to survey intrinsic fluorescence of epidermal cells collected from touch surfaces and investigate whether this property could potentially be used to discriminate between contributor cell populations in a biological mixture.  Analysis of red autofluorescence (650-670nm) showed that some contributors could be distinguished on this basis. Variation was also observed between autofluorescence profiles of epidermal cell populations from a single contributor sampled on different days. This dataset suggests that red autofluorescence may be a useful marker for identifying distinct cell populations in some mixtures. Future efforts should continue to investigate the extrinsic or intrinsic factors contributing to this signature, and to identify additional biomarkers that could complement this system.

## Introduction

The difficulties associated with interpreting complex DNA mixtures are well known in the forensic community, and are becoming more prevalent with the sharp increase in ‘touch’ or trace samples among forensic laboratories’ caseloads ^1^. Differentiating cell populations from individual contributors in a biological mixture before DNA analysis is a potential way to overcome this issue. While strategies exist to selectively label cell populations from distinct contributors based on their immunochemistry and then physically isolate cells from the mixture prior to DNA profiling ^2– 4^, there is a dearth of studies demonstrating cell separation techniques on touch samples. This is likely due to the fact that cell populations in these samples mostly, if not entirely, consist of fully differentiated keratinocytes which have limited reactivity to common molecular probes used to target surface antigens ^5, 6^.

An alternative approach is to avoid the need for probe binding by harnessing the intrinsic fluorescence of compounds found in or on epidermal cells. Here we report on our analysis of autofluorescence in the red region of the spectrum (650–670nm) of epidermal cells collected from surfaces touched by seven different individuals across multiple days, and the implications this may have for processing complex biological mixtures in forensic casework.

## Methods

Touch samples were collected from seven volunteers using the following protocol which was approved by the VCU-IRB (#HM20000454_CR). Volunteers rubbed a sterile polypropylene conical tube (P/N 229421; Celltreat Scientific) for five minutes using their entire hand (i.e., palm and fingers). Cells were collected from the surface with six sterile pre-wetted swabs (P/N 22037924; Fisher Scientific) followed by two dry swabs. To elute the cells into solution, the swabs were manually stirred then vortexed for 15 seconds in 10 mL of ultrapure water (18.2 MΩ∙cm). The entire solution was then passed through a 100 µm filter mesh prior to flow cytometry. Flow cytometry analysis of eluted cells was performed on the BD FACSCanto™ II Analyzer (Becton Dickinson) equipped with 488 nm and 633 nm lasers and a 660/20 nm detector filter. Channel voltages were set as follows: Forward Scatter (FSC, 150V), Side Scatter (SSC, 200V) and Allophycocyanin (APC, 250V). FSC and SSC channels were used to gate intact corneocytes for subsequent autofluorescence analysis. Gating of cell populations and generation of histogram profiles for each contributor was performed using FCS Express 4.0 Flow Research Edition (De Novo Software, Inc.).

## Results and discussion

**Figure 1.**
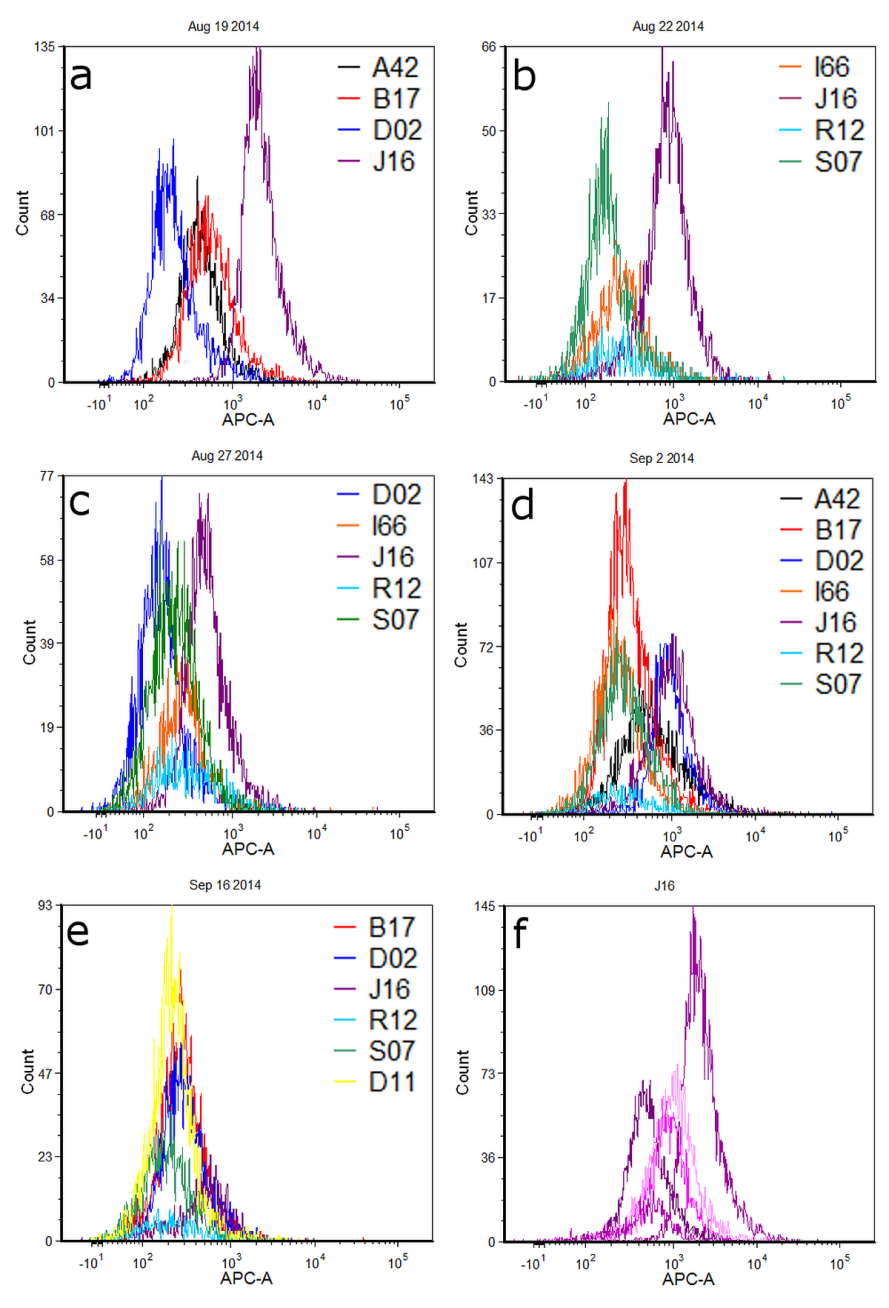
Overlayed red fluorescence channel histograms for epidermal cell populations from touch samples. Panels **a**– **e** show different combinations of donors cell populations each sampled and analyzed on the same day. Figure 1f is a histogram overlay of cell populations from contributor J16 across five different experiments.

Flow cytometry source data for individual contributors Flow Cytometry Standard (.fcs) format files are labeled by the corresponding panel in Figure 1 and the Donor ID. Click here for additional data file. Copyright: © 2016 Stanciu CE et al. 2016 Data associated with the article are available under the terms of the Creative Commons Zero "No rights reserved" data waiver (CC0 1.0 Public domain dedication).

Fluorescence histograms of individual cell populations from different donors are shown in Figure 1. For ease of comparison and visualization, profiles have been overlayed and grouped by the day on which cells were deposited, collected, and analyzed by flow cytometry. Clear differences in the red fluorescence (APC) channel are observed between several pairs of donor cell populations, particularly J16-D02 during the first experiment and J16-S07 in the second experiment ( Figures 1a and 1b respectively; Table 1). Most experiments resulted in one or more contributor cell population(s) whose fluorescence profile(s) could be distinguished from the others collected that day, such that a fluorescence intensity gate could be designed that would be expected to capture that contributor’s cells to the exclusion of (or minimal contribution of) cells from other contributors. However, significant and/or complete overlap was observed between many donor pairs (e.g., A42-B17 in Figure 1a; I66-S07 in Figure 1d). Sometimes, overlap of fluorescence distributions was such that gating could potentially separate the contributors into two or more groups (e.g. Figure 1d: A42, B17, I66, R12 and S07 in one group; D02 and J16 in another group). All contributors from the final experiment exhibited overlapping fluorescence histograms ( Figure 1e).

**Table 1.** Fluorescence histogram statistics for contributor cell populations ^1^.

Fig 1a					Fig 1b			
*Donor*	*Mean*	*Median*	*# Events* ^2^		*Donor*	*Mean*	*Median*	*# Events*
A42	540	427	3903		I66	341	253	1573
B17	743	556	4625		J16	996	842	3375
D02	305	212	5158		R12	497	252	599
J16	2606	2024	6475		S07	236	177	2497
Fig 1c					**Fig 1d**			
*Donor*	*Mean*	*Median*	*# Events*		*Donor*	*Mean*	*Median*	*# Events*
D02	208	160	3653		A42	959	554	4320
I66	372	276	1983		B17	409	307	7727
J16	635	491	3767		D02	1114	907	3524
R12	469	298	1090		I66	314	244	5014
S07	279	226	3751		J16	1245	982	4702
					R12	457	260	861
					S07	376	277	4676
Fig 1e					Fig 1f			
*Donor*	*Mean*	*Median*	*# Events*		*Donor*	*Mean*	*Median*	*# Events*
B17	349	280	3665		J16a	2606	2024	6475
D02	362	287	3041		J16b	635	491	3767
J16	589	515	1156		J16c	589	515	1156
R12	302	208	493		J16d	996	842	3375
S07	259	190	2028		J16e	1245	982	4702
D11	276	220	4230					

^1^Data is organized according to the histogram overlays shown in Figure 1. Mean (arithmetic) and median values are in relative fluorescent units (RFUs).

^2^Flow cytometry cell ‘events’ correspond to populations within FSC and SSC gates that select for intact epidermal cells.

Cell populations from J16 and D02 showed a great deal of disparity in fluorescence intensity in the first experiment, such that overlap between these populations was minimal ( Figure 1a). There was somewhat less distinction – and thus more overlap – observed between the same contributors during a second replicate ( Figure 1c); during a third, overlap between the two populations was substantial ( Figure 1d). As these results suggest, fluorescence intensity values for cell populations derived from any given contributor varied in distribution across replicate experiments on different days. Figure 1f shows overlayed histograms for J16 cell populations; mean fluorescence intensity values ranged from 589 to 2606 relative fluorescence units (RFUs) across five sampling days ( Table 1).

The underlying cause of red autofluorescence in these epidermal cell samples is currently unclear. Cells deposited through touch are likely primarily derived from the outermost epidermal layer (stratum corneum) which can contain a number of fluorescent compounds including tryptophan and tyrosine ^7, 8^, melanin, keratins, NADH and flavins ^9^, lipofuscins ^10^, and porphyrins and porphyrin precursors ^11, 12^. However, many of the corresponding emission maxima for these molecules occur at shorter wavelengths than what was examined in this study (e.g., amino acids, keratin, NADH, all have maxima below 550nm ^9^). Porphyrin molecules exhibit emission maxima between 630–680nm ^11^. Their abundance within the epidermis may be influenced by bacteria on the skin that produce porphyrin molecules with similar fluorescence emission profiles ^13^. Exogenous sources such as plasticides ^14^ or other biological compounds (e.g., chlorophyll ^15^) may also produce fluorescence, and could potentially be transferred to donors’ hands and subsequently to the tube surface (with cells) through touch or contact.

Regardless of the ultimate source for the observed differences in cell population fluorescence, this initial data set indicates that autofluorescence may be a useful marker for distinguishing between cell populations in a mixture. The non-destructive nature of flow analysis and the fact that autofluorescence monitoring does not require special reagents beyond those maintained in any laboratory (e.g. no probes required) are advantages when considering their potential front-end use in forensic analyses.

The variation across multiple samples from the same donor suggests that the level of autofluorescence is likely not a unique or identifying feature for a particular individual. However, to be of use in separating components of a biological mixture, a feature need not be unique; it simply needs to be distinctive among the contributors to that particular mixture. The ability to separate out even one contributor (or to separate a mixture of four contributors into two mixtures of two) may render the remaining mixture more interpretable in downstream DNA analysis. Further, the possibility that some combination of endogenous and/or exogenous factors could impart distinct optical properties to contributor cell populations in a particular mixture sample warrants further exploration.

Future efforts will continue to focus on isolating the molecule(s) responsible for fluorescent differences in touch epidermal cells through a combination of targeted immunofluorescent assays, chemical characterizations, and complex spectral analysis of autofluorescent profiles. Additionally, we are working on using optical signatures such as these to facilitate physical isolation of epidermal cell populations using flow cytometry-based strategies such as fluorescent activated cell sorting (FACS) for the purposes of generating single source genetic profiles from touch mixtures. Although previous work suggests that analyzing DNA profiles directly from isolated epidermal cells may be a challenge due to the prevalence of extracellular or ‘cell-free’ DNA in touch samples ^16^, the sheer quantity of cells that may be recovered from these sample types (up to ~1×10 ^5^, ^16^) may help to overcome such obstacles.

## Data availability

The data referenced by this article are under copyright with the following copyright statement: Copyright: © 2016 Stanciu CE et al.

Data associated with the article are available under the terms of the Creative Commons Zero "No rights reserved" data waiver (CC0 1.0 Public domain dedication). 

F1000Research: Dataset 1. Flow cytometry source data for individual contributors, 10.5256/f1000research.8036.d113749 ^17^
