# Supplementary material for: Analysis of red autofluorescence (650-670nm) in epidermal cell populations and its potential for distinguishing contributors to 'touch' biological samples
Source: F1000Res. 2016 Feb 16;5:180. [Version 1] doi: 10.12688/f1000research.8036.1 (PMC5130068; doi:10.12688/f1000research.8036.1)
Supplement: Peer review history

# Peer review: Referee response for version 1

**DOI:** 10.5256/f1000research.8645.r18017

**Published:** 2016-11-28

**Authors:** Mark Perlin

**Affiliations:** 1 Cybergenetics, Pittsburgh, PA, USA

The article’s title and abstract are appropriate. The design, methods and analysis have been explained, and are appropriate for this preliminary report. The conclusions are supported by the results. Data have been provided. Some context and suggestions for analysis are provided here.  

 Separation of cellular genetic material is central to forensic DNA analysis. Separation stages include:

1. Cells. Labs *separate* cells out of mixed cellular populations based on physical characteristics. For example, differential extraction can enrich sperm in one cell fraction relative to epithelial cells in another fraction. The red autofluorescence described in the paper occurs at this front-end stage. 
2. DNA. Extraction procedures *separate* nucleic acids from other molecules. Such procedures include organic, non-organic, chelex, FTA or silica methods.
3. Loci. PCR *separates* short DNA regions from the rest of the genome by amplifying (i.e., purifying) these specific regions relative to background sequence.
4. Fragments. Fluorescent electrophoresis *separates* DNA fragments by size, identifying length polymorphism alleles and determining their relative quantity.
5. Genotype. Computer *separation* of electrophoretic data can determine the genotypes of each contributor to a DNA mixture ^1^. Back-end methods that do not separate contributors ^2^ have limited applicability for resolving complex DNA mixtures. 

 

 This paper focuses on the first step, front-end separation of mixed epithelial cell populations. The authors investigate a novel physical assay – red autofluorescence in the 650-670 nm range. Their preliminary results demonstrate effective separation, with some cell populations clearly distinguished from others based on fluorescent frequency. 

 There are some limitations to the current procedure. The partial autofluorescence separation is incomplete, and can vary for the same subject between samplings. However, as the authors note, “the ability to separate out even one contributor [subset] may render the remaining mixture more interpretable in downstream DNA analysis.” This ability, coupled with automatic fluorescent activated cell sorting (FACS) and automatic DNA mixture data analysis (e.g., TrueAllele® computing), could be quite powerful. 

 Joint Bayesian analysis of multiple STR samplings of low-level DNA mixtures can recover considerable identification information ^3^. The autofluorescence method reported here could produce analogous data by taking FACS samplings at different fluorescent frequencies. Joint computer analysis of these different frequency-sampled STR amplifications would then complete the (mathematical) mixture separation into informative (probabilistic) genotypes. 

 The authors propose a novel front-end DNA separation method. As with most front-end methods, the cell separation is incomplete. However, their autofluorescence separation can become more complete if used in conjunction with other front-end discrimination methods. Moreover, combining their front-end cell separation with available back-end computer data separation ^1^^,^^3^ could extract considerable identification information from complex DNA mixtures.

I have read this submission. I believe that I have an appropriate level of expertise to confirm that it is of an acceptable scientific standard.

## References

[1] PerlinMWLeglerMMSpencerCESmithJLAllanWPBelroseJLDucemanBW: Validating TrueAllele® DNA mixture interpretation. *J Forensic Sci*.2011;56(6) : 10.1111/j.1556-4029.2011.01859.x 1430-47 10.1111/j.1556-4029.2011.01859.x 21827458

[2] BudowleBOnoratoAJCallaghanTFDella MannaAGrossAMGuerrieriRALuttmanJCMcClureDL: Mixture interpretation: defining the relevant features for guidelines for the assessment of mixed DNA profiles in forensic casework. *J Forensic Sci*.2009;54(4) : 10.1111/j.1556-4029.2009.01046.x 810-21 10.1111/j.1556-4029.2009.01046.x 19368620

[3] BallantyneJHansonEKPerlinMW: DNA mixture genotyping by probabilistic computer interpretation of binomially-sampled laser captured cell populations: combining quantitative data for greater identification information. *Sci Justice*.2013;53(2) : 10.1016/j.scijus.2012.04.004 103-14 10.1016/j.scijus.2012.04.004 23601717

# Peer review: Referee response for version 1

**DOI:** 10.5256/f1000research.8645.r16701

**Published:** 2016-10-28

**Authors:** Timothy J Verdon

**Affiliations:** 1 Victoria Police Forensic Services Department, Melbourne, VIC, Australia

The authors have investigated skin cell analysis by FACS in forensic biological analysis, and present some results which shed some light on the potential, or lack thereof, for this kind of technique. The experiment is technically sound and well presented, and although there is no statistical weight placed on the differences or similarities among cell species, some trends are obvious.

 Some minor amendments and areas for future examination would improve the paper:

- It must be noted earlier on (i.e. at the end of the first introductory paragraph) that much of the DNA in touch samples will be exogenous - the use of cell sorting for these kinds of samples may not actually yield appropriate quantities of DNA for profiling.
- The methodology of collecting touch samples raises some questions - the swab type used may have an effect on the cell structure and elution of the sample, possibly even may affect / introduce autofluorescence in some cases. Also were the swabs dried post-sampling or eluted straight afterwards? In actual casework this may also affect the quality of the keratinocytes, as will the lag between sampling and analysis. It would be of benefit to repeat this study with older samples, perhaps placed onto a swab and dried for a week before analysis, compared to pristine cells collected via a glass bead method ^1^  without the need for swabs at all.
- The cells collected and analysed herein may not all be borne of the individual who touched the item - how controlled were the volunteers before touching? Did they have to wash their hands? Did they have contact with others or other work environments? The only way to assess this fully would be to examine the profiles from these samples post-analysis.
- Following this, the samples may not actually yield sufficient DNA, as rightly pointed out in the discussion. It seems a shame that these samples were not separated and profiled to examine this further.

Overall, the publication of this type of data should be encouraged, and hopefully more thorough experiments of this kind will follow on from this work.

I have read this submission. I believe that I have an appropriate level of expertise to confirm that it is of an acceptable scientific standard.

## References

[1] QuinonesIDanielB: Cell free DNA as a component of forensic evidence recovered from touched surfaces. *Forensic Sci Int Genet*.2012;6(1) : 10.1016/j.fsigen.2011.01.004 26-30 10.1016/j.fsigen.2011.01.004 21292581
